# Selenium Supplementation Mitigates Copper-Induced Systemic Toxicity via Transcriptomic Reprogramming and Redox Homeostasis in Mice

**DOI:** 10.3390/foods14203528

**Published:** 2025-10-16

**Authors:** Faiz Hussain Panhwar, Muhammad Zahir Ahsan, Xiaomei Jia, Xiaoying Ye, Rongjun Chen, Lihua Li, Jianqing Zhu

**Affiliations:** 1 Rice Research Institute, Sichuan Agricultural University, 211, Huimin Road, Wenjiang District, Chengdu 611130, China; 2 Demonstration Base for International Science and Technology Cooperation of Sichuan Province, 211, Huimin Road Wenjiang District, Chengdu 611130, China

**Keywords:** selenium, copper toxicity, transcriptomics, redox homeostasis, heavy metal detoxification, murine model

## Abstract

Copper is an essential trace element that supports numerous physiological functions; however, excessive copper accumulation can disrupt cellular and biological processes. In this study, forty-eight male mice were randomly divided into four groups (n = 12): Control (fed normal rice), Cu300 (300 mg/kg copper), Cu300+Se (Cu300 + selenium-enriched rice), and Cu300+iSe (Cu300 + 1 mg/kg iSe), and were treated for 180 days. Copper exposure resulted in reduced body weight, hepatomegaly and nephritis, elevated copper deposition in organs, oxidative stress, and significant declines in RBC, HGB, and WBC counts, leading to anemia and immunosuppression. Selenium supplementation, effectively mitigated these effects by reducing copper accumulation, restoring antioxidant balance, and enhancing selenoprotein-related functions. Histopathological analysis revealed that copper toxicity induced hydropic degeneration and focal necrosis in hepatic and renal tissues, effects that were significantly attenuated by selenium supplementation. Transcriptomic profiling revealed that selenium-enriched rice reversed copper-induced gene expression changes. In the liver, selenium treatment significantly upregulated protective genes such as *Slc7a*, *Bola1*, *Uqcrq*, *Dtx1*, and *Znrd2*, while downregulating stress-related genes like *Trim75*, *Dpm3*, *Moxd1*, *Tnfrsf25*, and *Gpr75*. In the kidneys, selenium enhanced the expression of detoxification and immune-modulating genes (*Mt1*, *Mt2*, *Rhbdl1*, *Crisp3*, *Mif*) and suppressed stress-related genes (*Nnt*, *Ifi44l*, *NLRP12*, *Eno1b*, *Ugt1a*), demonstrating its role in mitigating oxidative and inflammatory stress. Collectively, these findings demonstrate that selenium-enriched rice exerts potent protective effects against chronic copper toxicity through multiple mechanisms: (1) restoration of mitochondrial function, (2) attenuation of ER stress and apoptosis, (3) enhancement of antioxidant and detoxification pathways, and (4) modulation of metabolic and immune responses. This study highlights selenium-enriched rice as a promising nutritional intervention for mitigating chronic copper toxicity and maintaining hepatorenal health.

## 1. Introduction

Copper (Cu), an essential trace element, plays a key role in numerous physiological functions, including cellular energy production, antioxidant defense, intracellular signaling, and the activity of various enzymes [1]. It serves as a cofactor for several critical enzymes, such as cytochrome c oxidase, lysyl oxidase, and superoxide dismutase [2]. However, excessive copper—whether due to environmental contamination or metabolic dysregulation—can accumulate in tissues, leading to oxidative stress, mitochondrial dysfunction, and inflammation, particularly in highly metabolic organs like the liver and kidneys [3].

The recommended daily intake of copper ranges from 0.3 to 0.7 mg and its systemic levels are tightly regulated through coordinated physiological processes. These include absorption in the duodenum and small intestine, transport via the bloodstream, hepatic storage and release, and elimination through bile [4]. Proper copper regulation is critical, as imbalances in copper homeostasis have been implicated in neurodegenerative diseases, metabolic disorders, and genetic conditions [5].

According to the World Health Organization (WHO), the maximum permissible concentration of copper in drinking water is 2 mg/L. However, due to increasing industrial activities, copper levels in some water sources may exceed 100 mg/L, or even reach 1000 mg/L [6]. This raises significant public health concerns and highlights the urgent need for effective strategies to mitigate copper toxicity.

The liver plays a central role in copper metabolism, overseeing its absorption, storage, and biliary excretion. Chronic copper overload can disrupt hepatic function, alter gene expression, and lead to hepatocellular injury [7]. Similarly, although less extensively studied, the kidneys are also susceptible to copper-induced oxidative stress, which may impair renal filtration and cause nephrotoxicity [8].

Selenium (Se), another essential trace element is involved in the regulation of more than 30 selenoproteins that contribute to antioxidant defense, immune modulation, and even cancer prevention [9,10]. Selenium helps maintain redox homeostasis, protects cells from oxidative injury, and can counteract the toxicity induced by heavy metals. In cadmium-exposed mice, for example, selenium has been shown to reduce liver damage, enhance antioxidant enzyme activity, and boost free radical scavenging capacity [11]. Nonetheless, genome-wide transcriptional changes to selenium supplementation during copper-induced stress remain poorly understood.

Selenium, particularly in the form of selenium-enriched diets, has demonstrated protective effects against copper-induced oxidative stress, suggesting a counteractive role against Cu toxicity [12]. Additionally, selenium influences copper metabolism by modulating its distribution across tissues and promoting detoxification through the induction of metallothioneins [13]. However, selenium’s efficacy depends on its dose, chemical form, and duration of exposure. At excessive levels, selenium itself can become toxic, indicating that the copper-selenium interaction is a finely tuned therapeutic balance [14,15]).

The liver, as the primary regulator of copper homeostasis, is often the first organ affected by copper toxicity, exhibiting histopathological changes, altered enzyme activity, and oxidative stress. The kidneys, responsible for reabsorption and excretion of trace elements, are also vulnerable to copper overload. While biochemical and histological studies have explored these organ-specific effects, the broader systemic molecular response to chronic copper exposure—especially at the transcriptomic level—remains inadequately characterized.

In this context, selenium-enriched rice represents a promising functional food approach to deliver bioavailable selenium in a safe, nutritionally balanced form. Unlike inorganic selenium supplements, selenium-enriched rice provides selenium organically bound within amino acids (such as selenomethionine), enhancing absorption efficiency and reducing toxicity risk. Moreover, rice is a global dietary staple, making selenium biofortification a practical and sustainable nutritional strategy to counteract trace metal toxicity.

Therefore, in the present study, we investigated the protective potential of selenium-enriched rice and inorganic selenium against chronic dietary copper exposure (300 mg/kg Cu diet) in mice. Using high-throughput RNA sequencing (RNA-seq), we examined transcriptomic alterations in the liver and kidneys to elucidate the molecular mechanisms of copper toxicity and selenium-mediated protection. By integrating differential gene expression and pathway enrichment analyses, this study aims to provide mechanistic insights into trace element interactions, identify potential biomarkers of Cu-induced stress and Se protection, and highlight the nutritional significance of selenium-enriched functional foods in mitigating heavy metal toxicity.

## 2. Materials and Methods

### 2.1. Chemicals and Reagents

Copper chloride dihydrate (CuCl_2_·2H_2_O), analytical grade (≥99.0% purity), was supplied by Sigma-Aldrich (St. Louis, MO, USA). Sodium selenite (Na_2_SeO_3_), pharmaceutical secondary standard (≥98.0% purity), was also procured from Sigma-Aldrich (St. Louis, MO, USA). Selenium-enriched rice (containing selenium 0.058 mg/kg) was provided by the International Demonstration Base for Rice Research, Sichuan Agricultural University (Chengdu, China). All other chemicals and solvents used were of standard analytical grade and were obtained from Sinopharm Chemical Reagent Co., Ltd. (Beijing, China).

### 2.2. Animals and Treatment

A total of 144 male Kunming mice (SPF grade, average weight 16 g), divided equally across three experimental replications, were obtained from Dashou Experimental Animals Company (Chengdu, China). Mice were housed with free access to food and water. After 1 week of acclimatization, they were randomly assigned into four groups, with 12 mice per group. Details of group allocation, diet, and copper/selenium treatments are provided in Table 1. Clinical observations—including grooming behavior, food and water intake, and social interactions were recorded daily at 10:00 AM. Body weight was measured weekly. During the study, each mouse was provided with 5 g of food daily and had free access to drinking water. At days 90 and 180, six mice from each group were randomly selected and euthanized under anesthesia. Organs were excised, washed, blotted dry, weighed immediately, and used to calculate the organ index (organ weight/body weight ratio). Blood samples were collected from the ocular vein. For serum collection, blood was allowed to clot at room temperature for 2 h and centrifuged at 3000 rpm for 10 min. Whole blood was also collected into EDTA-coated tubes for hematological analysis. All experimental procedures complied with institutional ethical guidelines for the care and use of laboratory animals.

### 2.3. Antioxidant Assay

Liver and kidney tissues were homogenized and centrifuged to obtain supernatants, which were then used to measure antioxidant indices. Levels of superoxide dismutase (SOD), glutathione peroxidase (GSH-Px), ferric reducing antioxidant power (FRAP), and malondialdehyde (MDA) were determined using commercial assay kits from Suzhou Keming Biotechnology Co., Ltd. (Suzhou, China), according to the manufacturer’s instructions. Spectrometric readings were taken using a Shimadzu UV-visible spectrophotometer (Model T6S, Puxi Co., Ltd., Beijing, China). Detailed assay protocols are available via the links provided in Table 2.

### 2.4. Histopathological Analysis

The left kidney and left lobe of the liver were fixed in 4% paraformaldehyde for histopathological evaluations. Tissues were sectioned and stained with hematoxylin and eosin (H&E) for microscopic examination, following the method described by (Su et al., 2021 [11]).

### 2.5. Hematological Analysis

Hematological parameters were measured in blood samples collected at days 90 and 180. Samples were transferred into EDTA-coated tubes to prevent clotting and stored at 4 °C until analysis. Measurements were performed using the Sysmex K4500 Hematology Analyzer (Sysmex Corporation, Kobe, Japan), according to the manufacturer’s guidelines. For each sample, 50 µL of whole blood was analyzed in triplicate to ensure data reliability.

### 2.6. Quantification of Copper and Selenium in Organs

Copper and selenium concentrations in liver, kidney, spleen, and lungs were determined using an atomic fluorescence spectrophotometer (AFS, Model RGF-6800, Bo Hui Co., Ltd., Beijing, China). The analysis followed the procedure described by (Su et al., 2021 [11]). Elemental concentrations (mg/kg) were calculated using the formula:

$$Contents=\frac{\left(C\;-\;Co\right)\;\times \;V}{m\;\times \;1000}$$

where:

C is the measured concentration of the sample solution (µg/L)

C_o_ is the concentration in the control group (µg/L)

m is the mass of the samples (g)

V is the total volume of the digested solution (mL)

### 2.7. Quantitative Real-Time PCR (qRT-PCR) Analysis

Liver and kidney samples were rinsed with pre-chilled DEPC-treated water to remove RNase contamination. About 100 mg of each tissue was pulverized in liquid nitrogen using a sterile mortar and pestle. Total RNA was extracted with TRIzol reagent (Takara, Ichikawa, Japan) according to the manufacturer’s instructions. The RNA was then reverse-transcribed into complementary DNA (cDNA) using a kit from Vazyme (Nanjing, China), which served as the template for qRT-PCR.

qRT-PCR was performed as described by Su et al., 2021 [11], using GAPDH as the internal control gene for normalization. Primer sequences are listed in Table S1 (Supplementary Materials). Gene expression was quantified using the 2^−ΔΔCT^ method. Each reaction was conducted with three biological replicates and two technical replicates to ensure reproducibility. Primers were designed using NCBI Primer-BLAST to ensure specificity and amplification efficiency. All qRT-PCR analyses were conducted using standardized procedures to minimize variability and enhance data reliability.

### 2.8. Transcriptome Analysis

The samples from liver and kidneys were taken from mice supplemented with 300 mg kg^−1^ food Cu (CK) and 300 mg kg^−1^ food Cu and selenium-enriched rice (Cu300+Se).

### 2.9. RNA Extraction and Quality Control, Library Preparation, and Sequencing

Total RNA was extracted from the liver and kidney tissues using TRIzol reagent following the manufacturer’s protocol. RNA purity and concentration were assessed using a Nano Drop spectrophotometer (Thermo Fisher Scientific, Waltham, MA, USA) and RNA integrity was verified by agarose gel electrophoresis. RNA-Seq libraries were prepared using the TruSeq Stranded mRNA library Prep Kit (Illumina, San Diego, CA, USA) followed by the Poly-A selection. Libraries were quantified by qPCR and sequenced on an Illmuina HiSeq/NovaSeq platform to generate 150 bp paired-end reads.

### 2.10. Bioinformatics Analysis

Raw data were quality checked using FastQC (v0.11.9). Adapters and low quality reads were trimmed using Trimmomatic (v0.39) or Cutadapt (v4.4). Clean reads were aligned to the mouse reference genome (GRCm38/mm10) using HISAT2 (v2.2.1) or STAR (v2.7.10a). Differentially expressed genes (DEGs) between control and treatment were identified using DESeq2 (v1.38.3) where |Log2FC| > 2 and adjusted *p*-value < 0.05 was considered. Gene Ontology (GO) and Kyoto Encyclopedia of Gens and Genomics (KEGG) pathway analyses were conducted using cluster profiler (v4.6.2) and DAVID (v2023q4).

### 2.11. Statistical Analysis

The experimental data were analyzed using IBM SPSS statistics (Version 27) (IBM Corp., Armonk, NY, USA, 2020) and Microsoft Excel (Microsoft Corporation, Redmond, WA, USA, 2019). Data are presented as mean ± SD. Difference between groups were assessed by one-way ANOVA followed by Tukey’s post-hoc test (*p* < 0.05). Data visualization and graph plotting were performed using GraphPad Prism version 10.0.0 for Windows (GraphPad Software, Boston, MA, USA, www.graphpad.com).

## 3. Results

### 3.1. Effect of Selenium Supplementation on Body Weight and Organ Coefficient

**Figure 1:**
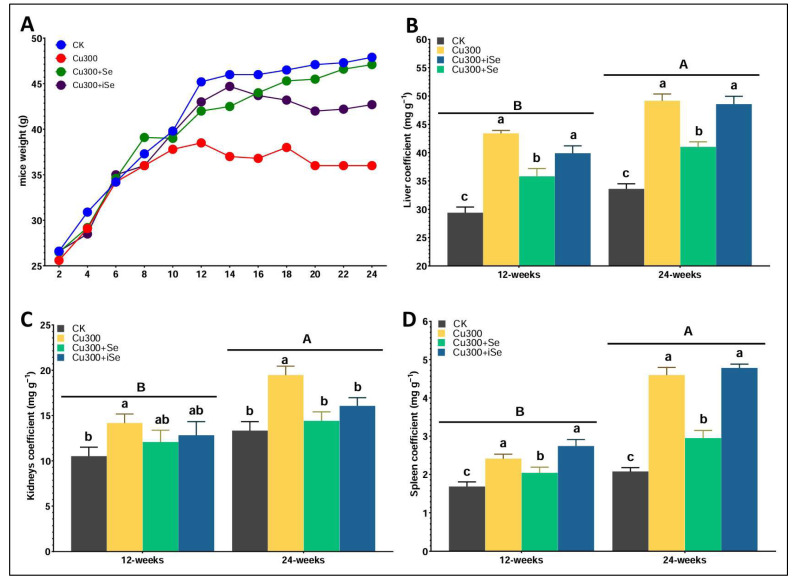
The biweekly body weight (**A**) and organ coefficients of liver (**B**), kidneys (**C**), and spleen (**D**) across treatment groups at 90 and 180 days of exposure. Data are presented as mean ± standard deviation where n = 6 lowercase letter above the bars indicate a significant difference among the treatment groups and capital letters indicate the difference between time-points. Statistical analysis was performed using one-way ANOVA, with significance at *p* < 0.05.

Mice exposed to copper (Cu) exhibited significantly reduced weight gain over 24 weeks compared to the control (CK) and selenium-supplemented groups (Figure 1A). Both selenium treatments (Cu300+Se and Cu300+iSe) mitigated Cu-induced weight loss, with the selenium-enriched rice group (Cu300+Se) showing nearly normal weight gain by 24 weeks. At both 90 and 180 days, Cu exposure significantly increased the liver coefficient compared to the control (Figure 1B). Selenium supplementation, especially in the form of selenium-enriched rice (Cu300+Se), effectively reduced liver enlargement at 90 days. By 180 days, both selenium forms resulted in similar liver weights, significantly lower than those in the Cu-only group. At 90 days, Cu exposure significantly elevated the kidney coefficient relative to the control (Figure 1C). Selenium supplementation partially reversed this effect, resulting in intermediate values. By 180 days, although Cu continued to cause kidney enlargement, both Cu300+Se and Cu300+iSe treatments maintained slightly reduced coefficients, indicating a protective trend with selenium. Cu treatment also increased the spleen coefficient at both time points (Figure 1D). Selenium supplementation significantly attenuated this effect at 90 days and brought spleen coefficients close to control levels by 180 days. Both selenium forms were effective in counteracting Cu-induced spleen hypertrophy. Overall, Cu toxicity negatively impacted weight gain and caused hypertrophy in the liver, kidneys, and spleen. Selenium supplementation, particularly in its organic form, consistently alleviated these adverse effects, highlighting its protective role against Cu-induced organ stress and toxicity.

### 3.2. Copper and Selenium Deposition in Different Organs

**Figure 2:**
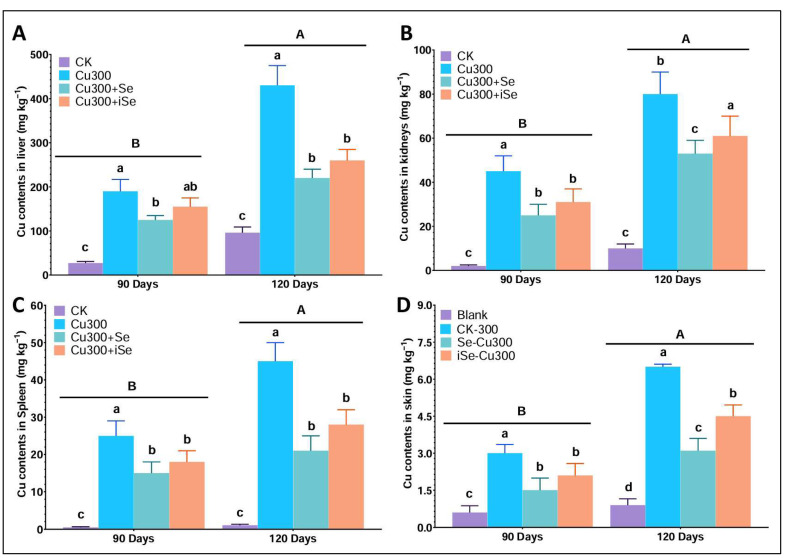
Cu contents in liver (**A**), kidneys (**B**), spleen (**C**), and skin (**D**) of mice across different treatments at 90 and 180 days of exposure. Data are presented as mean ± standard deviation where n = 6 lowercase letter above the bars indicate significant difference among the treatment groups and capital letters indicate the statistical difference between the time points. Statistical analysis was performed using one-way ANOVA, with significance at *p* < 0.05.

**Figure 3:**
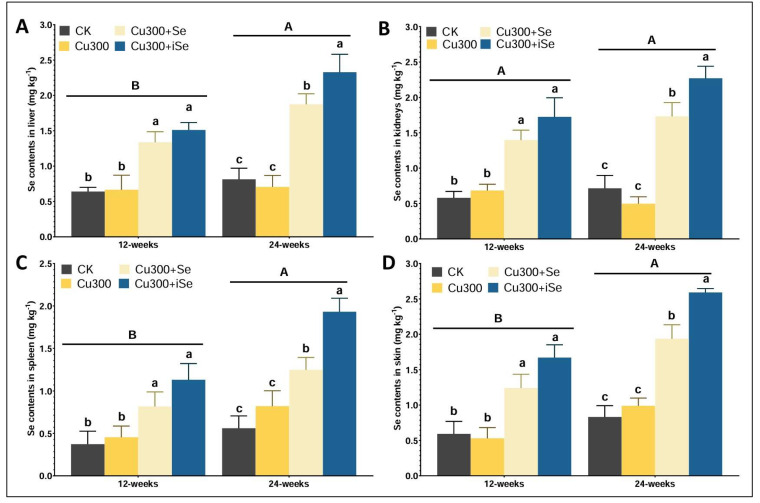
Selenium contents in liver (**A**), kidneys (**B**), spleen (**C**), and skin (**D**) of mice across different treatments at 90 and 180 days of exposure. Data are presented as mean ± standard deviation where n = 6 lowercase letter above the bars indicate significant difference among the treatment groups and capital letters indicate the statistical difference between the time points. Statistical analysis was performed using one-way ANOVA, with significance at *p* < 0.05.

Copper accumulation in mouse organs varied significantly across treatment groups and time points (Figure 2). Under Cu300 treatment, tissue Cu levels reached potentially toxic concentrations, particularly in the liver (441 mg/kg) and kidneys (85.9 mg/kg) at 180 days (Figure 2A,B). These values exceeded known toxicity thresholds, indicating a risk of hepatic and renal damage. Cu300 also elevated Cu levels in the spleen (43 mg/kg) and skin (6.25 mg/kg), reflecting systemic copper overload (Figure 2C,D). Selenium-enriched rice (Cu300+Se) significantly reduced Cu accumulation in all organs, lowering liver Cu to 228 mg/kg and kidney Cu to 53.3 mg/kg. Inorganic selenium (Cu300+iSe) also decreased Cu levels, with liver Cu reduced to 290 mg/kg and kidney Cu to 63.5 mg/kg. Both selenium forms attenuated Cu accumulation, with organic selenium demonstrating slightly stronger detoxifying effects, particularly at 180 days. These reductions indicate selenium’s protective role in mitigating chronic Cu toxicity. Selenium accumulation in mouse organs was significantly influenced by the form of selenium supplementation under copper exposure (Figure 3). Cu300 treatment alone did not markedly increase Se levels compared to the control, with only marginal changes observed across organs at both time points. In contrast, Cu300+Se significantly elevated Se levels, especially in the liver (1.37 mg/kg) and kidneys (1.73 mg/kg) at 90 days (Figure 3A,B). This trend persisted at 180 days, although the levels remained slightly lower than those observed in the inorganic Se group. The Cu300+iSe treatment (1 mg/kg inorganic selenium) led to the highest Se accumulation in all organs at 180 days: liver (2.26 mg/kg), kidneys (2.37 mg/kg), skin (2.39 mg/kg), and spleen (1.93 mg/kg). Selenium levels were consistently higher in the Cu300+iSe group compared to Cu300+Se, indicating greater bioaccumulation. Among all organs, the liver and kidneys showed the most pronounced Se accumulation. These findings demonstrate that both selenium forms enhance tissue Se content under Cu exposure. Notably, the Se concentrations observed in the Cu300+iSe group at 180 days approached or slightly exceeded the 2.0–2.4 gm/kg range, which may be near the threshold for chronic selenium toxicity, particularly with prolonged exposure or in sensitive tissues.

### 3.3. Selenium Supplementation Effects on Endogenous Antioxidant Enzymatic Activity

**Figure 4:**
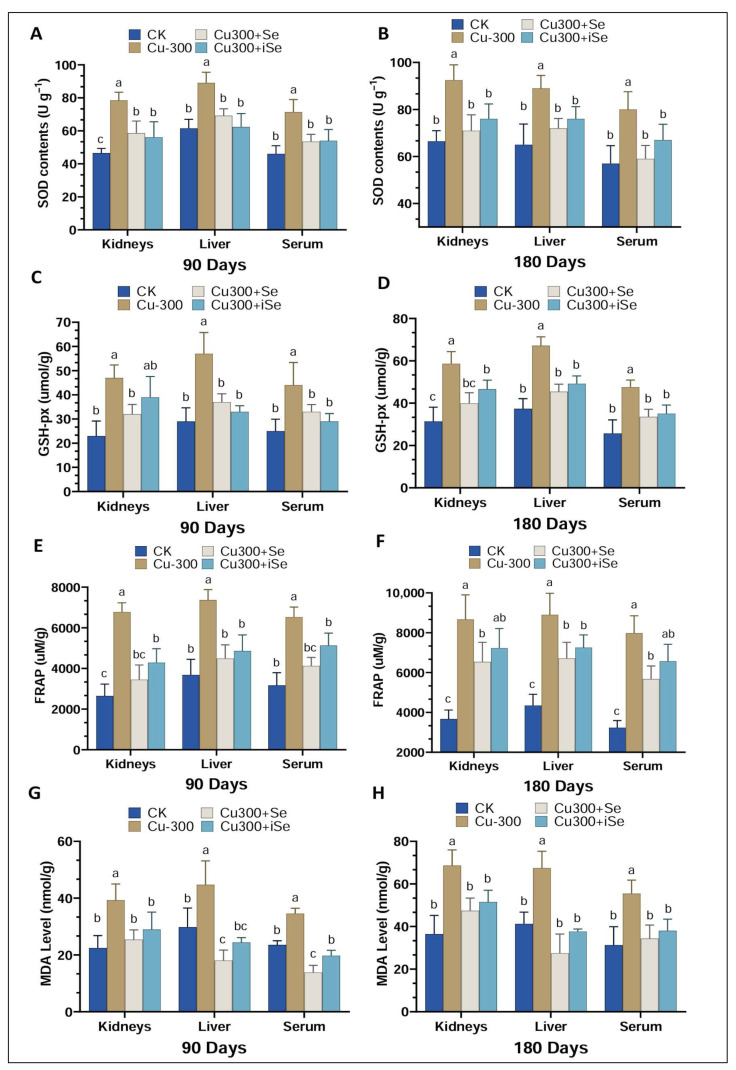
Superoxide dismutase (SOD), GSH-Px, TAOC, and MDA levels in the liver, kidneys, and serum of mice at 90 and 180 days of exposure. (**A**), SOD concentration at 90 days, (**B**), SOD concentrations at 180 days, (**C**) GSH-px concentrations at 90 days, (**D**) GSH-px concentrations at 180 days, (**E**) FRAP concentrations at 90 days, (**F**) FRAP concentrations at 180 days, (**G**) MDA concentrations at 90 days, (**H**) MDA concentrations at 180 days. Data are presented as mean ± standard deviation where n = 6 lowercase letter above the bars indicate significant difference among the treatment groups. Statistical analysis was performed using one-way ANOVA, with significance at *p* < 0.05.

Superoxide dismutase (SOD) activity varied significantly across tissues (kidney, liver, and serum) and treatment groups, reflecting tissue-specific and time-dependent responses to Cu and selenium exposure (Figure 4A,B). At 90 days, SOD activity was elevated in all tissues of the Cu300 group compared to the control. Both selenium treatments (Cu300+Se and Cu300+iSe) attenuated this increase. By 180 days, SOD activity remained elevated in the Cu300 group, with organic selenium showing a stronger normalizing effect than inorganic selenium. Similarly, glutathione peroxidase (GSH-Px) activity increased in the kidneys and liver of Cu-treated mice at both time points, GSH-Px) but selenium co-treatment restored activity to near control levels (Figure 4C,D). Total antioxidant capacity, measured by ferric reducing antioxidant power (FRAP), also increased following Cu exposure (Figure 4E,F), but was significantly reduced with selenium supplementation. Malondialdehyde (MDA) levels, a marker of lipid peroxidation and oxidative stress, were elevated in all tissues of the Cu300 group at both 90 and 180 days (Figure 4G,H). Selenium supplementation effectively lowered MDA levels though responses varied by tissues. All antioxidant parameters (SOD, GSH-Px, FRAP, and MDA) showed more pronounced changes at 180 days compared to 90 days, indicating cumulative oxidative stress and adaptive antioxidant responses over time. These findings demonstrate that copper exposure induces oxidative stress and elevates antioxidant enzyme activity, while selenium—particularly in its organic form—offers protective effects by counteracting these disturbances in a time- and tissue-dependent manner.

### 3.4. Copper and Selenium Supplementation Changes the Hematological Parameters

Chronic copper exposure (Cu300) resulted in marked hematological alterations, indicative of systemic toxicity and impaired hematopoiesis (Table 3). At both 90 and 180 days, Cu300 significantly reduced red blood cell (RBC) count, hemoglobin (HGB), and hematocrit (HCT) levels, consistent with anemia. Additionally, reductions in mean corpuscular volume (MCV) and mean corpuscular hemoglobin (MCH) were more pronounced at 180 days, suggesting the development of microcytic hypochromic anemia. White blood cell (WBC) counts and lymphocyte (Lym) numbers were also suppressed in the Cu300 group, indicating potential immunosuppression and bone marrow toxicity.

Selenium supplementation, particularly in its organic form (Cu300+Se), showed partial to substantial recovery of several hematological parameters. At 90 days, organic Se restored RBC, HGB, and HCT values closer to control levels and improved immune cell counts (WBC, neutrophils, and lymphocytes). By 180 days, Cu300+Se maintained higher RBC and HGB levels than the Cu300 group, although values remained below control, indicating partial but sustained protection. In contrast, inorganic Se (Cu300+iSe) demonstrated mixed efficacy. While it improved WBC and Neu counts and moderately alleviated anemia at 90 days, it was less effective by 180 days, with RBC, HGB, and HCT values significantly lower than both the control and Cu300+Se groups.

Platelet (PLT) levels were notably elevated in the Cu300 group at 180 days, likely reflecting a compensatory response to oxidative damage or inflammation. Selenium supplementation partially normalized these platelet counts, with organic Se again demonstrating superior effectiveness. Overall, these findings indicate that chronic Cu exposure impairs hematopoietic function, leading to anemia and immunosuppression. Selenium supplementation—especially in organic form—confers significant protective effects on blood parameters.

### 3.5. Copper and Selenium Supplementations Affect the Normal Architecture of the Liver and Kidneys

**Figure 5:**
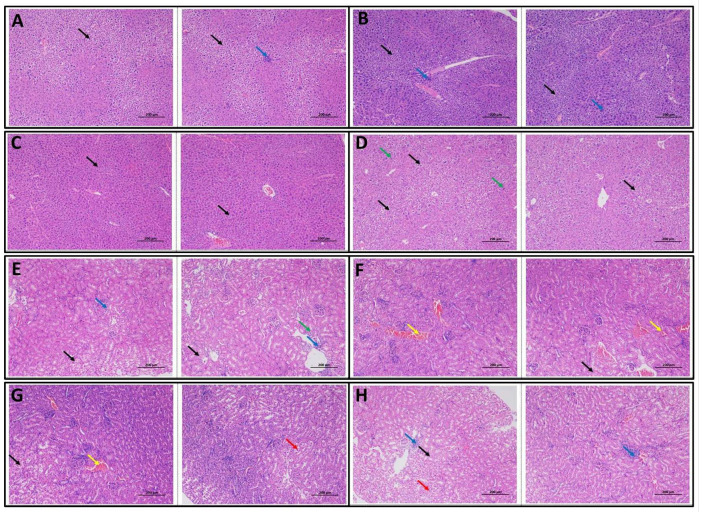
Histopathological analysis of liver and kidneys under copper stress and selenium supplementation. (**A**) Liver control, (**B**) liver Cu300, (**C**) liver Cu300+Se, (**D**) liver Cu300+iSe, (**E**) kidneys control, (**F**) kidneys Cu300, (**G**) kidneys Cu300+Se, and (**H**) kidneys Cu300+iSe.

In the liver tissues of the control group (Control Liver) (Figure 5), a large number of hepatocytes exhibited moderate hydropic degeneration characterized by cell swelling and pale, lightly stained cytoplasm (black arrows). Occasional hepatocyte necrosis was observed in the parenchyma, marked by loss of structural integrity and infiltration of surrounding lymphocytes (blue arrows). In the Cu300 group (Cu300 Liver), many hepatocytes showed mild hydropic degeneration, with loose, pale-stained cytoplasm, and swelling (black arrows). Occasional necrotic hepatocytes were present within the hepatic parenchyma, accompanied by localized lymphocytic infiltration (blue arrows). In the copper and selenium-enriched rice group (Cu300+Se), hepatocytes exhibited slight hydropic degeneration (black arrows), including visible cytoplasmic vacuolation and pale staining, but no significant necrosis or inflammatory cell infiltration was observed. In copper and inorganic selenium group (Cu300+iSe), numerous hepatocytes showed moderate hydropic degeneration (black arrows), with pale-stained cytoplasm and cellular swelling; nuclei varied in size, and a few mitotic figures were observed (green arrows) but without notable necrosis or inflammation.

In the kidneys of the control group (Control Kidneys), the glomeruli were evenly distributed with uniform cell numbers and matrix components. A few renal tubular epithelial cells showed mild hydropic changes (black arrows), including swelling and pale cytoplasm. Occasional, connective tissue hyperplasia around interstitial blood vessels was noted, along with minimal lymphocyte infiltration (blue arrow). Tubular atrophy was occasionally observed, identified by reduced volume and irregular shape (green arrow). In the kidneys of copper group (Cu300 kidneys), the glomeruli remained evenly distributed with consistent cellular and matrix appearance. Some tubular epithelial cells displayed mild hydropic degeneration (black arrows) and rare instances of interstitial vascular congestion were observed (yellow arrows), though no significant hyperplasia or inflammation was observed. In the kidneys of copper and selenium-enriched rice group (Cu300+Se), the glomeruli and matrix appeared uniform and a small number of tubular epithelial cells exhibited mild hydropic degeneration (black arrows). Occasionally, necrosis and detachment of cells were noted (red arrows) and rare interstitial vascular congestion was seen (yellow arrows) without evident hyperplasia or inflammation. In the kidneys of copper and inorganic selenium group (Cu300+iSe kidneys), the glomeruli were evenly distributed with a uniform matrix. Some tubular epithelial cells exhibited mild hydropic changes (black arrows), a small number showed necrosis and detachment (red arrows). Mild lymphocytic infiltration was occasionally observed around the interstitial vessels (blue arrows).

### 3.6. Copper and Selenium Supplementation Alter the Transcriptional Factors of the Liver and Kidneys

**Figure 6:**
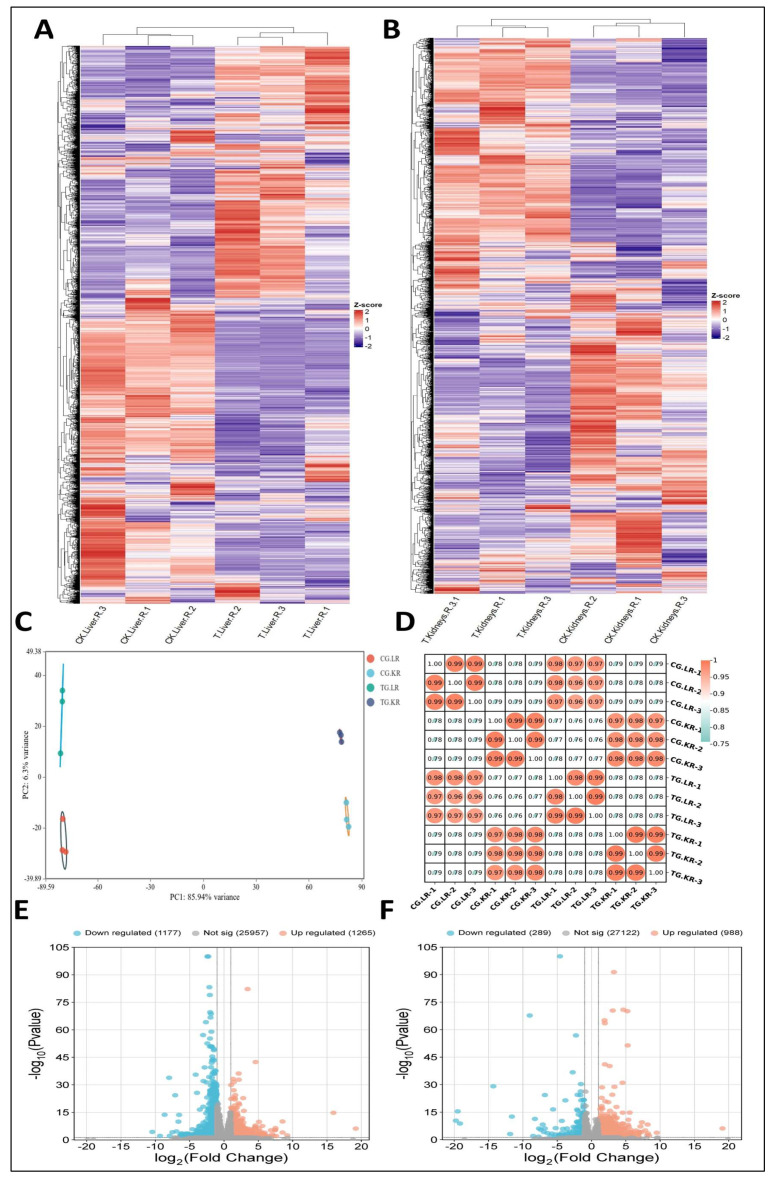
Transcriptome analysis of two treatments. Overview of liver (**A**) and kidney (**B**) transcriptome (**C**) principal components analysis of samples, (**D**) correlation heatmap of DEGs, (**E**) volcano map of DEGs of treatment vs control in liver, and (**F**) volcano map of DEGs of treatment vs control in liver. See Tables S2 and S3 for liver and kidneys DEG details.

To understand how selenium-enriched rice alleviates the copper stress, we performed transcriptome analysis of liver and kidney tissues to uncover the gene regulatory network. A total of 3033 DEGs in liver and 1883 differential genes in kidneys were detected between the control and treatment groups (Figure 6A,B; Tables S2 and S3). The correlation analysis revealed significant differences between groups and replications within the groups clustered together, indicating the data’s reproducibility (Figure 6D). Principal component analysis also demonstrated that all groups were well separated from each other and replications clustered together on principal component 1 explaining 85.9% variation between samples, demonstrating that the data were well structured (Figure 6C). Using the criteria log2FC > |1| and FDR < 0.01, we identified 2442 DEGs in liver, of which 1265 were upregulated and 1177 were downregulated, and 1277 DEGs in kidneys, of which 988 were upregulated and 289 were downregulated. The results showed that more genes were upregulated when selenium-enriched rice was given along with copper (Figure 6E,F).

### 3.7. Functional Characterization of DEGs Associated with Copper Stress

**Figure 7:**
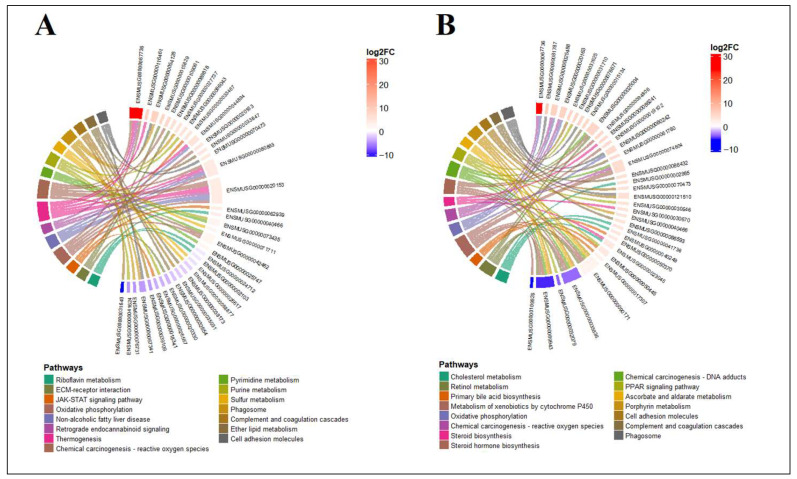
KEGG enrichment of liver (**A**) and kidneys (**B**) in treatment vs control. The top three genes with the most significant changes in each of the top 10 pathways are listed in the figure.

To assess the functional role of differentially expressed genes (DEGs) in response to copper stress, we conducted a KEGG pathway enrichment analysis identifying the top 15 most significantly enriched pathways in the liver and kidneys (Figure 7A,B). In the liver, metabolic stress pathways (ko00190, ko04932, ko00230, ko00240, ko04714, ko00565), oxidative stress and detoxification pathways (ko05208, ko00740, ko00920), inflammatory and immune response pathways (ko04630, ko04610, ko04145), structural and extracellular matrix pathways (ko04512, ko04514), and signaling pathways (ko04723) were prominently enriched.

Key observations in the liver included upregulation of oxidative phosphorylation (47 DEGs), chemical carcinogenesis-reactive oxygen species (53 DEGs), and thermogenesis (57 DEGs), with fewer downregulated genes. In contrast, riboflavin metabolism (2 upregulated, 7 downregulated) and purine metabolism (11 upregulated, 20 downregulated) showed more balanced or inverse trends. In the kidneys, enriched pathways included metabolic and detoxification process (ko00830, ko00980, ko00860, ko00053, ko04979, ko00100), oxidative stress and carcinogenesis (ko05204, ko05208), energy metabolism (ko00190), inflammatory and immune response (ko04610, ko04514, ko04145), hormonal and signaling (ko03320, ko00100) and bile acid and lipid homoeostasis (ko00120) (Table S4). Notably, chemical carcinogenesis–ROS (38 upregulated, 6 downregulated) and oxidative phosphorylation (31 upregulated, 2 downregulated) exhibited strong upregulation, while retinol metabolism (8 upregulated, 15 downregulated) and steroid hormone biosynthesis (7 upregulated, 12 downregulated) displayed more downregulation. Overall, the majority of DEGs were upregulated, suggesting a robust transcriptional activation in response to copper stress, with tissue-specific variations in pathway regulation.

### 3.8. Pathway Level Analysis of Copper- and Selenium-Induced Molecular Responses

**Figure 8:**
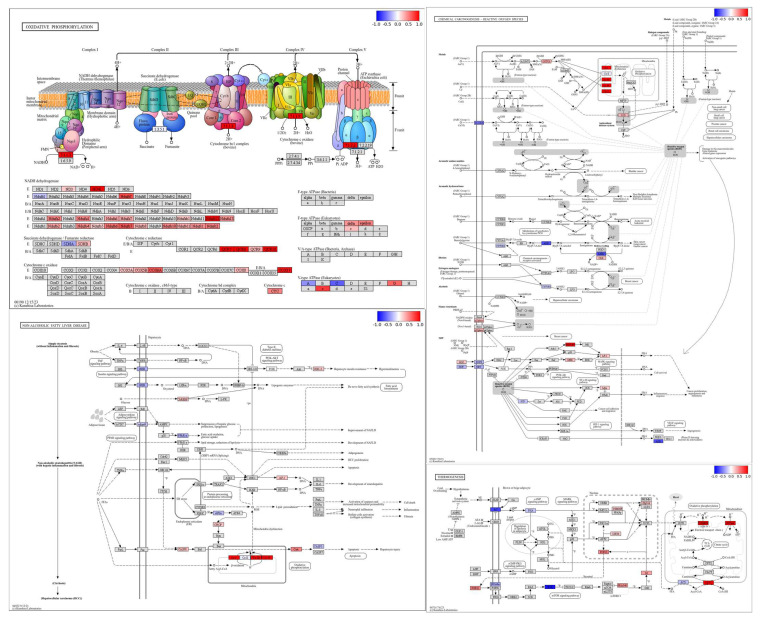
Main pathways related to stress under treatment (Cu300+Se) and control treatments (Cu300) in liver.

**Figure 9:**
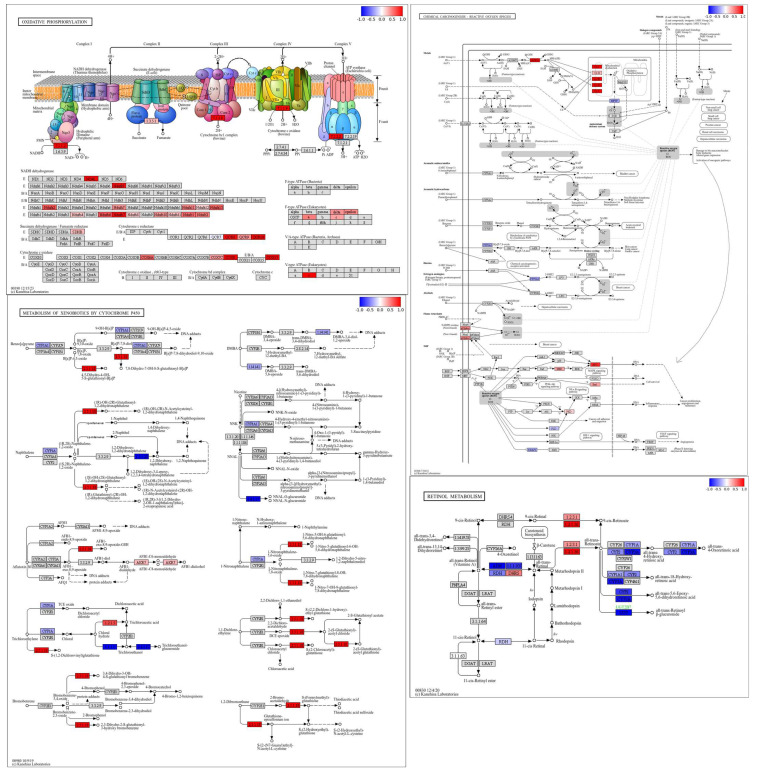
Main pathways related to stress under treatment (Cu300+Se) and control treatments (Cu300) in kidneys.

Under copper stress, key components of the oxidative phosphorylation pathway, especially in Complex I (ND4L, Ndufs7, Ndufa2, Ndufa4, Ndufa6, Ndufa7, Ndufa12, Ndufa13, Ndufb4, Ndufb6, Ndufb7, Ndufb8, Ndufb10) and Complex III (Cyc1, Uqcrc1, Uqcrc2), showed strong downregulation (Figure 8). Selenium supplementation with selenium-enriched rice helped recover the expression of these components, particularly in Complexes I, III, IV, and V. The F-type ATPase subunit also showed partial restoration, improving the energy production potential. Overall, selenium-enriched rice significantly alleviated copper-induced impairment of oxidative phosphorylation. Furthermore, selenium enriched rice treatment reactivated the critical thermogenic genes, particularly UCP1, and stress-related genes like SIRT1 and FGF21, indicating improved stress response and mitochondrial biogenesis. Some upstream regulators, such as PRAS40 and SWI/SNF, remained moderately suppressed. Overall, selenium mitigated copper-induced inhibition of thermogenesis and boosted mitochondrial efficiency. Under copper stress, critical components of the non-alcoholic fatty liver disease (NAFLD) pathway, including GSK3-β, Chop, CASP8, and mitochondrial complexes, were strongly downregulated, indicating enhanced ER stress, apoptosis and mitochondrial dysfunction. Selenium-enriched rice treatment partially restored insulin signaling (via INSR), reduced ER stress markers, and suppressed apoptotic signals like CASP8 and CytC. Protective regulators PPAR-α and PPAR–γ were upregulated, supporting lipid metabolism recovery. Although mitochondrial oxidative phosphorylation improved, full restoration was incomplete. Overall, selenium-enriched rice mitigated copper-induced NAFLD progression and liver cell injury (Figure 9). In the kidneys, components of the oxidative phosphorylation pathway showed marked upregulation. Several subunits in Complex I (NADH dehydrogenase), including ND4L, Ndufa2, Ndufa7, Ndufa11, Ndufa12, Ndufa13, Ndfub4, Ndufb6, Ndufb7, Ndufb8, Ndufb10, Ndufb11, Ndufc1, and Ndufc2, were highly upregulated. Complex II (succinate dehydrogenase) showed mild activity with SDHB slightly upregulated. In Complex III (cytochrome bc1 complex), subunits such as QCR8, QCR9, and QCR10 were notably upregulated. Complex IV (cytochrome c oxidase) exhibited strong upregulation of COX6A, COX7C, COX3, and COX17. Finally, Complex V (ATP synthase) showed increased expression of the alpha, beta, delta, and c subunits, suggesting enhanced ATP production activity. In the retinol metabolism pathway, enzymes involved in carotenoid biosynthesis (such as those associated with 1.2.3.1 and 1.2.1.36) were significantly upregulated. Conversely many cytochrome P450 enzymes (like CYP1A1, CYP3A, and CYP2 family members) were strongly downregulated, indicating reduced oxidative metabolism of retinoids. ADH and associated dehydrogenases also showed downregulation, suggesting suppressed conversion of retinol forms. Enzymes involved in retinyl ester formation, such as DGAT and LRAT, remained mostly unchanged. Overall, the results point to enhanced retinoid synthesis but reduced retinoid catabolism. In the metabolism of xenobiotics by cytochrome P450 pathway, several glutathione S-transferase enzymes (notably associated with EC 2.5.1.18 and EC 2.5.1.13) were highly upregulated, suggesting enhanced detoxification activity. Cytochrome P450 enzymes like CYP3A7 and CYP2E1 showed notable upregulation in certain branches. In contrast, enzymes like CYP1A1, CYP1B1, and CYP2A6 were downregulated, indicating reduced activation of some toxic intermediates. Various conjugation pathways, especially glutathione conjugation, were strongly activated. Overall, the pathway reflects a shift toward detoxification and reduced xenobiotic bioactivation (Figure 9).

### 3.9. Validation of Transcriptomic Data Through qRT-PCR

**Figure 10:**
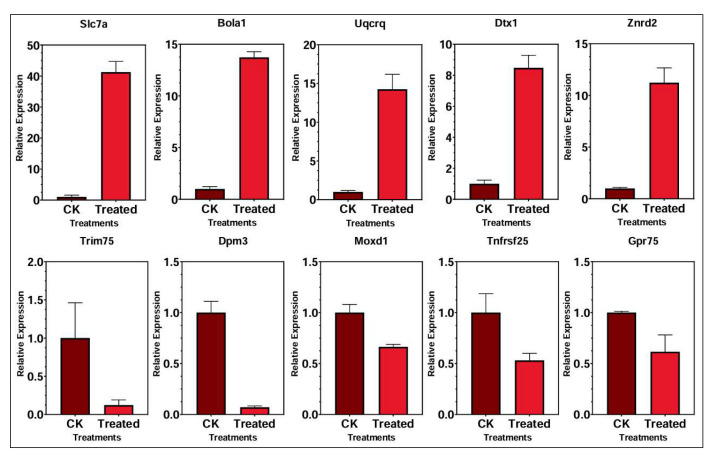
Major up- and downregulation genes in the liver in treatment (Cu300+Se) control (Cu300) groups.

The relative expression levels of ten genes in the liver, as validated through qRT-PCR analysis (Figure 10). Among these, five genes—*Slc7a*, *Bola1*, *Uqcrq*, *Dtx1*, and *Znrd2*—were significantly upregulated in the selenium-enriched rice treated group compared to the control. Notably, *Slc7a* exhibited the highest induction, with expression increasing nearly 40-fold following treatment. Similarly, *Bola1* and *Uqcrq* showed substantial increases of approximately 13- to 15-fold. *Dtx1* and *Znrd2* also showed substantial upregulation, with relative expression levels exceeding 8- and 10-fold, respectively. In contrast, five genes—*Trim75*, *Dpm3*, *Moxd1*, *Tnfrsf25*, and *Gpr75*—were significantly downregulated in the treated liver samples. *Trim75* and *Dpm3* demonstrated the most pronounced repression, with expression reduced to near-baseline levels. The other downregulated genes (*Moxd1*, *Tnfrsf25*, and *Gpr75*) also showed significant decline in mRNA expression compared to the control, indicating treatment-induced transcriptional suppression.

**Figure 11:**
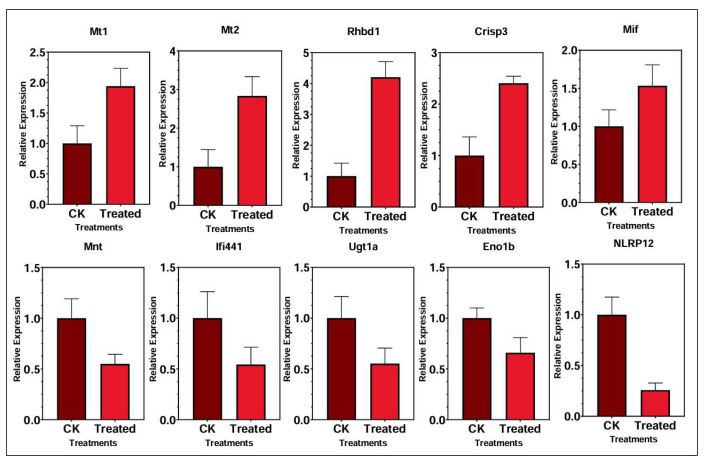
Major up- and downregulation genes in the kidneys in treatment (Cu300+Se) control (Cu300) groups.

Figure 11 presents the relative mRNA expression levels of ten genes in the kidney following treatment, as validated via qRT-PCR. Five genes—*Mt1*, *Mt2*, *Rhbdl1*, *Crisp3*, and *Mif*—were significantly upregulated in the selenium-enriched rice group compared to the control. *Mt2* exhibited the highest induction, with expression level increasing more than threefold. *Rhbdl1* and *Crisp3* also showed substantial increase of approximately fourfold and twofold, respectively. *Mt1* and *Mif* were notably upregulated, suggesting a strong transcriptional response to selenium supplementation. Conversely, five genes—*Nnt*, *Ifi441*, *NLRP12*, *Eno1b*, and *Ugt1a*—were significantly downregulated in the selenium-treated group. Among these, *NLRP12* and *UGT1a* showed the most marked reduction, while *Nnt* and *Iffi44l* also exhibited significant decline in expression in the treatment group compared to the control.

## 4. Discussion

Mice subjected to elevated copper levels (300 mg/kg) exhibited significant reductions in body weight and increases in liver, kidney, and spleen coefficients, indicating systemic toxicity and organ hypertrophy. These findings align with previous reports showing that excess copper induces oxidative stress, mitochondrial dysfunction, and tissue injury in mammals (Agency for Toxic Substances and Disease Registry (ATSDR) 2004). Selenium supplementation, particularly in its organic form, demonstrated protective effects against copper-induced toxicity. By 180 days, mice in the Cu300+Se group exhibited weight gain patterns similar to the control group, suggesting attenuation of copper’s detrimental effects on growth and metabolism. Organ coefficients were also lower in selenium-treated groups, especially at 90 days, indicating reduced organ hypertrophy. These observations are consistent with recent studies highlighting selenium’s antioxidative and cytoprotective properties in metal-induced toxicity models [16,17].

The superior efficacy of organic selenium over its inorganic counterpart may stem from differences in bioavailability and metabolic conversion. Organic selenium is more efficiently incorporated into functional selenoproteins such as glutathione peroxidase, which are essential for reducing reactive oxygen species (ROS) and maintaining redox balance [17]. In contrast, inorganic selenium may be less efficiently utilized and exhibit inconsistent protective effects.

This study underscores the complex interaction between copper and selenium under chronic exposure. High-dose copper treatment (Cu300) significantly elevated copper concentrations in the liver and kidneys, surpassing known toxicity thresholds and indicating a risk of hepatic and renal damage [3,18]. Selenium co-supplementation reduced copper accumulation, with both selenium-enriched rice (organic selenium) and sodium selenite (inorganic selenium) treatments attenuating copper levels. Organic selenium showed slightly greater efficacy at later time points. These findings support previous research indicating that selenium mitigates heavy metal toxicity via antioxidative and metal sequestration mechanisms [11,19].

However, selenium accumulation itself must be carefully monitored. Inorganic selenium led to higher tissue deposition approaching or exceeding levels (2.0–2.4 mg/kg) associated with potential chronic selenium toxicity [20]. This aligns with studies reporting a narrow margin between selenium’s nutritional requirement and its toxicity threshold, particularly under prolonged exposure. Copper and selenium interaction also influences selenoprotein regulation. Excess copper has been shown to impair the synthesis and trafficking of selenoprotein P (SELNOP), disrupting systemic selenium homeostasis [21]. Thus, selenium supplementation may help restore selenoprotein function while simultaneously protecting against copper overload. These findings highlight selenium’s therapeutic potential against chronic copper toxicity, though the form and dosage must be carefully optimized to balance detoxification efficacy with safety.

The variation in antioxidant enzyme activities and oxidative stress markers across different tissues and treatment durations reflects the complex interplay between copper-induced oxidative stress and selenium’s protective effects. Chronic exposure to elevated copper levels (Cu300) led to significant increases in superoxide dismutase (SOD) and glutathione peroxidase (GSH-Px) activities in the kidneys, liver, and serum at both 90 and 180 days, indicating an adaptive response to heightened ROS levels. Concurrently, elevated malondialdehyde (MDA) levels in all tissues confirmed lipid peroxidation and oxidative damage. These findings are consistent with studies demonstrating that excessive copper induces oxidative stress, mitochondrial dysfunction, and hepatotoxicity. For example, Li et al. [22] reported activation of the PERK/ATF4-mediated endoplasmic reticulum stress pathway in copper-exposed mice, contributing to liver damage.

In contrast, selenium supplementation mitigated copper-induced oxidative stress. Organic selenium, in particular, was more effective in restoring antioxidant enzyme activities and reducing MDA levels. This enhanced efficacy is likely due to its higher bioavailability and efficient integration into selenoproteins. Researchers reported similar protective effects of selenium-enriched rice in cadmium-exposed mice [11]. Selenium’s role in upregulating selenoproteins and maintaining redox balance is well established.

Hematological analysis revealed that chronic copper exposure (300 mg/kg) significantly disrupted blood homeostasis, indicating systemic toxicity and impaired hematopoiesis. Reduced RBC, HGB, and HCT levels in Cu300-treated mice are indicative of hypochromic microcytic anemia, consistent with previous findings that excess copper impairs erythropoiesis, disrupts iron metabolism, and induces oxidative stress in hematopoietic tissues [23,24]. Alterations in WBC and lymphocyte counts further suggest immune dysregulation, likely caused by copper-induced oxidative damage and bone marrow toxicity. High copper levels have been reported to exert cytotoxic effects on immune cells and alter cytokine expression, leading to immunosuppression [25]. Selenium supplementation attenuated these hematological abnormalities [26], with organic selenium being notably more effective. This may be due to its superior cellular uptake and incorporation into functional selenoproteins, such as glutathione peroxidase, which protect hematopoietic stem cells from ROS-induced damage [27]. In contrast, inorganic selenium (sodium selenite) demonstrated limited efficacy and higher risk of toxicity with prolonged exposure [28,29]. Elevated platelet counts in Cu300-treated mice may reflect oxidative injury or inflammation both of which were moderated by selenium, particularly in its organic form, further supporting its anti-inflammatory and antioxidative roles.

Histopathological analyses of liver and kidney tissues revealed that high-dose copper exposure induced varying degrees of cellular damage, primarily hydropic degeneration and occasional necrosis. Hydropic degeneration, characterized by cytoplasmic swelling due to ionic imbalance, is an early indicator of toxic insult [30]. Mild hydropic changes in the control group may reflect baseline stress, while reduced severity in the Cu300+Se groups supports selenium’s protective effect. Selenium is known to mitigate oxidative damage, stabilize cellular membranes, and enhance antioxidant defenses, particularly through selenoenzyme activity [31]. Copper, while essential, becomes toxic in excess, causing oxidative stress, mitochondrial dysfunction, and organ damage especially in the liver and kidneys [2,3]. Transcriptomic analysis in this study showed that copper exposure (300 mg/kg) disrupted gene expression in both organs, activating stress pathways and impairing metabolic functions. In the liver, copper exposure suppressed oxidative phosphorylation, particularly Complexes I and III, consistent with previous findings of impaired mitochondrial function under copper stress [32,33]. Downregulation of genes such as *ND4L*, *Ndufs7*, and *Uqcrc1* indicated mitochondrial dysfunction and energy deficiency. Additionally, activation of the NAFLD pathway and ER stress markers CHOP and CASP8 suggested progression toward liver injury and apoptosis [34]. Selenium-enriched rice supplementation reversed many of these transcriptomic changes. Expression of genes related to oxidative phosphorylation—including Complex I (Ndufa2, Ndufb8), Complex III (Uqcrc1, Cyc1), and Complex IV (Cox6A, Cox7C)—was restored. Selenium’s role in promoting mitochondrial biogenesis and protecting respiratory complexes through selenoproteins such as GPX and TrxR has been well documented [31,35]. Restoration of mitochondrial function likely contributed to improved energy metabolism. Additionally, selenium reactivated thermogenic and stress-related genes such as *UCP1*, *SIRT1,* and FGF21, indicating enhanced mitochondrial efficiency and stress adaptation. *SIRT1* activation is known to support mitochondrial biogenesis and protect against metabolic disease [36]. Upregulation of PPAR-α and PPAR-γ further supports selenium’s role in improving lipid metabolism and reducing inflammation under copper stress [37].

In the kidneys, copper exposure paradoxically upregulated oxidative phosphorylation pathways, possibly reflecting a compensatory stress response, to maintain ATP levels [38]. Selenium-supplementation enhanced the expression of mitochondrial complex subunits, suggesting a more effective restoration of mitochondrial respiration. Detoxification pathways were also modulated. Kidney expression of glutathione S-transferase (GST) enzymes increased, indicating enhanced xenobiotic detoxification (Flore et al. 2008), while cytochrome P450 enzymes (e.g., CYP1A1, CYP1B1) were downregulated, reducing toxic intermediate formation [39]. Selenium-induced promotion of phase II detoxifying enzymes is a known protective mechanism [40]. Retinol metabolism showed upregulation of synthetic enzymes and suppression of catabolic ones, possibly preserving vitamin A levels for epithelial and immune function maintenance under oxidative stress [41].

qRT-PCR validation confirmed major transcriptional alterations in liver and kidney tissues under Cu300 stress and their mitigation by selenium-enriched rice. In the liver, upregulation of *Slc7a*, *Bola1*, *Uqcrq*, *Dtx1*, and *Znrd2* reflected selenium’s protective role. *Slc7a* encodes a cationic amino acid transporter involved in metal detoxification and redox balance [42]. Enhanced expression of *Uqcrq* and *Bola1* supports mitochondrial function, while *Dtx1* and *Znrd2* are associated with transcriptional and zinc-finger stress responses [31]. Downregulation of genes such as *Trim75*, *Dpm3*, *Moxd1*, *Tnfrsf25*, and *Gpr75* highlights selenium’s attenuation of immune and stress signaling [27]. In the kidneys, upregulation of *Mt1*, *Mt2*, *Rhbdl1*, *Crisp3*, and *Mif* indicates enhanced detoxification and immune modulation. Metallothioneins (*Mt1*, *Mt2)* serve as biomarkers of metal stress [43], while downregulation of *Nnt*, *Ifi44l*, *NLRP12*, *Eno1b*, and *Ugt1a* reflects restored redox and immune balance [44,45].

The findings of this study have several important practical applications. In the realm of public health, the results strongly support the use of organic selenium, specifically in the form of selenium-enriched rice, as a dietary intervention for populations at risk of chronic copper exposure, whether from environmental contamination or occupational hazards. For the food and agricultural industry, this research provides a scientific basis for developing selenium-enriched rice as a novel functional food for human consumption and for formulating safer, more effective animal feed supplements. The inclusion of organic selenium in livestock feed could mitigate the adverse effects of copper supplements used as growth promoters, thereby enhancing animal welfare and reducing copper pollution in manure. Furthermore, our data on the differential efficacy and tissue accumulation of selenium forms offer valuable insights for regulatory bodies, informing more precise guidelines on the recommended form and dosage of selenium in nutritional supplements and fortified foods to maximize benefits and minimize the risk of selenotoxicity.

While this study provides comprehensive evidence for the protective role of organic selenium, certain limitations should be acknowledged. Firstly, the study was conducted in a controlled murine model and the translational applicability of the findings to other species or real-world exposure scenarios requires further validation. Secondly, although we identified transcriptomic changes, the specific contributions of individual selenoproteins in mediating the observed protection were not isolated, leaving the precise molecular mechanisms to be fully elucidated. Finally, the long-term consequences of the selenium intervention beyond 180 days remain unknown. Future research should therefore focus on applied trials in agriculturally relevant animals, utilize genetic knock-out models to pinpoint the roles of key selenoproteins, and investigate the lifelong health outcomes of such nutritional interventions to fully assess their efficacy and safety.

Overall, these findings underscore selenium-enriched rice’s robust protective effect against copper-induced toxicity. The main mechanisms include restoration of mitochondrial function, reduction of ER stress and apoptosis, enhancement of antioxidant and detoxification defenses, and modulation of key metabolic and immune pathways. Selenium-enriched rice may thus represent a promising nutritional strategy for mitigating copper toxicity and preserving hepatic and renal health. Future studies should further investigate the roles of individual selenoproteins and assess long-term outcomes of selenium intervention under chronic copper exposure.

## Figures and Tables

**Table 1:** Mice grouping, diet, and copper/selenium treatments.

Group	Diet	Cu Treatment	Se Treatment	No. of Mice
Control (CK)	Ordinary rice	0	0	12
Cu300	Ordinary rice	300 mg/kg	0	12
Cu300+Se	Se-enriched rice	300 mg/kg	0	12
Cu300+iSe	Ordinary rice	300 mg/kg	1 mg/kg	12

**Table 2:** Antioxidant assay protocol links.

Sr.	Assay Name	Protocol Link
1	FRAP	http://cominbio.com/uploads/soft/180727/1-1PHG53253.pdf (accessed on 24 May 2024)
2	SOD	https://www.cominbio.com/uploads/soft/210918/1-21091Q33444.pdf (accessed on 24 May 2024)
3	GSH-Px	https://cominbio.com/uploads/soft/180727/1-1PHGA647.pdf (accessed on 24 May 2024)
4	MDA	http://www.cominbio.com/uploads/soft/180727/1-1PHG64442.pdf (accessed on 24 May 2024)

**Table 3:** Hematological parameters measured at 90 and 180 days of exposure.

Parameter	90 Days				180 Days			
	CK	Cu300	Cu300+Se	Cu300+iSe	CK	Cu300	Cu300+Se	Cu300+iSe
WBC	4.59	3.91	4.94	4.87	5.03	3.81	4.78	2.37
Neu	0.76	0.35	0.83	0.95	1.39	0.99	1.40	1.76
Lym	3.16	3.17	3.7	3.44	3.06	1.16	1.95	1.07
Mon	0.47	0.25	0.29	0.27	0.47	0.51	0.24	0.44
Eos	0.2	0.14	0.12	0.2	0.11	0.14	0.09	0.10
Bas	0	0	0	0.01	0.00	0.01	0.00	0.00
Neu %	16.4	9	21.1	19.4	27.5	26.0	19.1	32.0
Lym %	68.9	81	68.5	70.5	60.9	56.7	64.7	44.9
Mon %	10.3	6.2	7.1	5.6	9.3	13.3	11.6	18.7
Eos %	4.4	3.6	3.2	4.2	2.3	3.7	4.6	4.2
Bas %	0	0.2	0.1	0.3	0.0	0.3	0.0	0.2
RBC	9.01	8.24	8.72	9.51	8.67	5.47	8.65	6.67
HGB	165	146	156	167	125	97	125	102
HCT	47.6	40.2	42.9	47.2	37.4	26.5	39.3	31.1
MCV	52.8	48.8	49.2	49.6	43.2	45.4	46.6	48.4
MCH	18.3	17.7	17.9	17.5	14.4	14.5	15.3	17.6
MCHC	346	362	363	353	335	319	328	365
RDW-CV	14.7	14.3	15.1	16	19.2	23.5	21.6	20.3
RDW-SD	35.9	32.3	34.4	36.9	39.1	50.1	47.3	45.9
PLT	539	246	286	285	590	716	472	392
MPV	7.4	7.9	6.6	6.4	6.4	6.6	6.3	6.0
PDW	16.2	17	16.4	16.8	15.4	16.3	15.5	15.4
PCT	0.4	0.193	0.189	0.183	0.378	0.472	0.296	0.235

## Data Availability

The original contributions presented in this study are included in the article/Supplementary Materials. Further inquiries can be directed to the corresponding author.

## Supplementary Materials

The following supporting information can be downloaded at: https://www.mdpi.com/article/10.3390/foods14203528/s1, Table S1: List of Primers used for qRT-PCR. Table S2: Differentially Expressed Genes of Mice Liver under Copper stress. Table S3: Differentially Expressed Genes of Mice Kidneys under Copper stress. Table S4: Enriched Pathways in Liver and kidneys of mice under copper stress.
